# Pulmonary manifestations in Niemann-Pick type C disease with mutations in *NPC2* gene: case report and review of literature

**DOI:** 10.1186/s12881-017-0367-x

**Published:** 2017-01-17

**Authors:** Jayesh Sheth, Jijo John Joseph, Krati Shah, Mamta Muranjan, Mehul Mistri, Frenny Sheth

**Affiliations:** 1 FRIGE’s Institute of Human Genetics, FRIGE House, Jodhpur Gam Road, Satellite, Ahmedabad, 380 015 India; 2 Department of Pediatrics, Believers Church Medical College Hospital, Tiruvalla, Kerala 689 101 India; 3 Department of Paediatrics, KEM Hospital, Parel, Mumbai, 400 012 India

**Keywords:** Alveolar proteinosis, Case report, Hepatosplenomegaly, Lung involvement, Lysosomal Storage Disorders, Niemann-Pick disease type C, *NPC2* gene

## Abstract

**Background:**

Niemann-Pick disease type C (NPC) is an inherited metabolic disorder; due to defect in cellular cholesterol trafficking. It is clinically a heterogeneous disease with variable age of onset with multiple organ systems being involved. *NPC1* gene is involved in 95% cases where as remaining ~5% cases are linked with *NPC2* gene.

**Case presentation:**

Case-1, a 14-months-old female presented with recurrent respiratory distress, failure to thrive and hepatosplenomegaly. Lung biopsy was suggestive of alveolar proteinosis and liver biopsy confirmed foamy macrophages. Molecular analysis revealed homozygous mutation c.141C > A in exon 2 of *NPC2* gene.

Case-2, a 3-year-old male presented with dyspnoea and hepatomegaly noticed at 1 year of age. HRCT-scan of thoracic region showed consolidation with mediastinal lymphadenopathy. Broncho-alveolar lavage revealed moderate amount of foamy macrophages and bone marrow examination detected foam cells. Homozygous T > C transition in intron 1 of the *NPC2* gene was identified.

**Conclusion:**

Our study demonstrates that NPC2 can present in early years of life with pulmonary complications like alveolar proteinosis and hepatosplenomegaly or hepatomegaly due to mutation in *NPC2* gene. An early suspicion will help clinicians to clinch its diagnosis, management and genetic counselling.

## Background

Niemann–Pick disease type C (NPC) is a fatal autosomal recessive neurovisceral disorder due to mutation in *NPC1* and *NPC2* genes leading to alterations in trafficking of endocytosed cholesterol [1]. Due to heterogeneous clinical phenotype, NPC is underdiagnosed and often missed altogether. In NPC, the protein product of the *NPC1* gene functions as a transporter of cholesterol and glycolipids in the endosomal-lysosomal system whereas the smaller protein product of NPC2 cooperates with the NPC1 protein [2, 3]. NPC2 plays a vital role in endosomal/lysosomal cholesterol trafficking by markedly accelerating the rates of transport from and between membranes [4]. The mechanism of NPC2 action involves direct interaction of the protein with membranes. The defects in NPC1 and NPC2 proteins, leads to sequestration of cholesterol derived products in the cell leading to hepatosplenomegaly, pulmonary and neurological manifestations [3]. *NPC1* gene is responsible for 95% of manifestations with main phenotype being hepatosplenomegaly and nervous system. The other ~5% of the NPC is caused by *NPC2* gene where pulmonary manifestations with respiratory failure have been documented [5]. Till date nearly twenty cases have been reported of NPC2 with twelve homoallelic mutations and none from India. Present study is the first report of *NPC2* from India with primary pulmonary manifestations and hepatosplenomegaly highlighting phenotype-genotype correlation.

## Case presentation

Case-1: 9 month old female child born to non-consanguineous parents from Rajasthan (Marwadi community) presented with recurrent respiratory tract infection and failure to thrive. She was frequently hospitalized for tachypnea and developed hepatosplenomegaly in next 5 months. Multiple treatment modalities were tried including antibiotics, nebulisations, anti-fungal agents, systemic corticosteroids and moist oxygen inhalation. In spite of all these, child remained tachypneic and continued to be oxygen dependent.

**Fig. 1:**
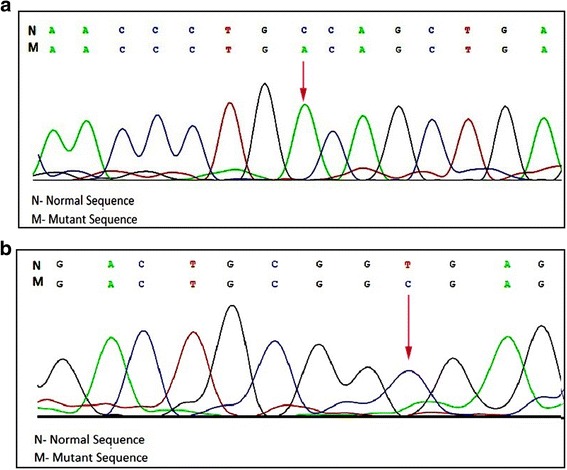
Sequencing of the NPC genes. **a** Chromatogram showing homozygous nonsense mutation c.141C > A (p.C47X) in exon 2 of *NPC2*. **b** Homozygous intronic c.82 + 2 T > C transition mutation detection in intron 1 of *NPC2*

Her chest radiogram showed hazy, diffuse bilateral alveolar infiltrates. HRCT showed ground glass opacification of the right upper lobe, suggestive of alveolar proteinosis. Lung biopsy with PAS staining showed dilated alveoli, lined by type-2 pneumocytes, containing foamy macrophages further indicating alveolar proteinosis. Possibility of a secondary alveolar proteinosis was kept in view of hepatosplenomegaly along with lung involvement. Liver biopsy showed fairly well preserved lobular architecture and cord pattern, few lymphocytes in portal tract and finely vacuolated ballooned hepatocytes and kupffer cell were suggestive of Niemann-Pick disease. Further studies revealed high plasma chitotriosidase level with fifty percent reduced activity of acid Sphingomyelinase [3.6 nmol/hr/mg – NR: 8.0-14.5 nmol/hr/mg protein]. This has clinched the clinical suspicion of NPC in the proband. Infilatration of the lungs with liver disease in the proband pointed towards NPC2 diagnosis. Therefore bi-directional sanger sequencing covering exon-intron boundries of *NPC2* gene was carried out. This has identified homozygous nonsense variant (c.141C > A) (p.Cys47Ter) in exon 2 (Fig. 1a). This variant is reported as pathogenic in HGMD (CM052291) and ClinVar (dbSNP80358263) database and responsible for protein truncation (variation ontology:0015).

Case-2: 1 year old male child born to third degree consanguineous parents from Maharshtra (Brahmin community) presented with dyspnoea, hepatomegaly and failure to thrive. Limbs were hypotonic with normal reflexes and power. Chest X-rays showed hyperinflation initially and progressive perihilar alveolar infiltration sparing peripheries. HRCT scan of thoracic region showed patchy alveolar fibrosis with mediastinal lymphadenopathy. Broncho-alveolar lavage revealed moderate amount of foamy macrophages and bone marrow examination showed presence of foamy histiocytic cells. Enzyme study for Niemann Pick type A, B and Gaucher’s disease were normal. Since acid sphingomyelinase was normal and pulmonary manifestations and hepatomegaly were predominantly present, NPC was suspected. Further study was carried out by bi-directional Sanger sequencing of *NPC2* gene covering exon-intron boundries. This has identified homozygous splice site variation c.82 + 2 T > C (g.5191 T > C) in *NPC2* gene (Fig. 1b). This variant is reported as pathogenic in HGMD (CS032706) and ClinVar (dbSNP:879253740) database that affect RNA splicing (variation ontology:0362).

## Discussion

NPC is a rare neurovisceral disease with an overall incidence of 1:150,000, of which only 5% cases are linked to NPC2 [6]. It is caused due to deposition of unesterified cholesterol mainly in brain, liver and lungs. Primary diagnosis of NPC by filipin stain is considered as the gold standard [7]. However, being tedious and time consuming, molecular testing was introduced in 2000 where the molecular defect was detected in *NPC1* and *NPC2* gene located on chromosome 18q11.2 and 14q24.3 respectively [3]. Since then several studies have shown an association of pulmonary manifestation and hepatosplenomegaly with NPC2 [8–11]. Likewise the two cases under study had a similar clinical presentation and were investigated primarily for acid sphingomyelinase (aSMase) activity in leucocytes. Case 1 had shown nearly 50% reduced activity of sphingomyelinase while case 2 had shown normal activity. Nearly fifty percent reduced activity of spingomyelinase in case 1 can be due to gross disruption of sphingomyelinase processing by intracellular cholesterol loading affecting aSMase activity as has been shown in CHO cell lines [12]. Pulmonary phenotype with reduced aSMase activity gave a clue of NPC2 in case 1. Identical phenotype led to consideration of NPC2 in the second case. Molecular study has identified previously known pathogenic non sense and RNA splicing mutations respectively. Both *NPC2* mutations are known to be associated with pulmonary manifestations which may be a result of loss of normal NPC2 protein expression in alveolar macrophages leading to an accumulation of functionally inactive surfactant rich in cholesterol [11].

In absence of specific biomarkers for NPC2, non-enzymatic plasma levels of certain cholesterol oxidation products (oxysterols) can be used for clinical screening of NPC. However, this screening method alone cannot distinguish between causative gene *NPC1* from *NPC2* [13].

*NPC1* and *NPC2* carrying mutants are identical in terms of pathogenesis and clinical phenotype/s but there lies a discrete demarcation where pulmonary manifestations give clue to the diagnosis of *NPC2* gene [14]. Identification of *NPC2* as the causative factor for NPC with pulmonary involvement was shown by Park et al. for the first time in 2003 [15]. Moreover, three mutations in the *NPC2* gene were subsequently notified by Verot et al. in 2007 [16]. In 2005, Chikh et al. demonstrated that missense mutation of *NPC2* causes misfolding of endoplasmic reticulum proteins [17].

**Fig. 2:**
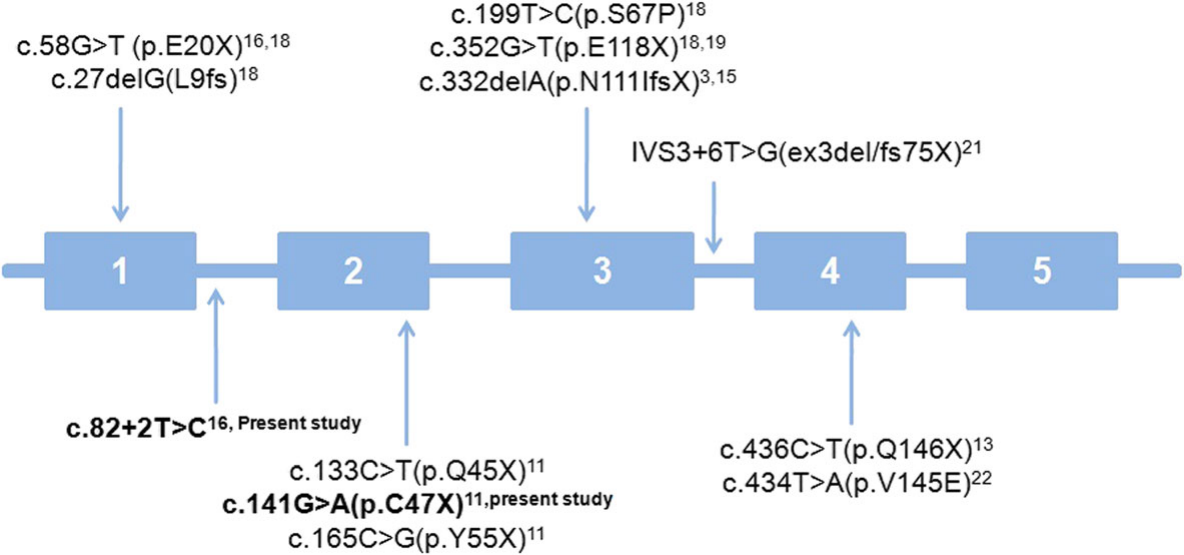
Schematic view of mutations reported in *NPC2* gene having pulmonary manifestation. Mutations observed in the present study are shown in bold

A detailed clinical analysis and mutation in *NPC2* have clearly shown pronounced lung involvement as a cause of early death in six patients as shown by Millat et al. and several others [18–20]. Similar severity was observed in the present cases. Till date, nearly twenty patients with twelve different homoallelic mutations in *NPC2* gene with pulmonary manifestations have been reported (Fig. 2, Table 1) [20, 21]. Documentation of these mutations with phenotypic presentation has become obligatory as commonly occurring bronchitis/pneumonia with liver involvement and failure to thrive are likely to mimic NPC2. Our study emphasizes the association of homoallelic mutation of *NPC2* gene with non responsive alveolar fibrosis/proteinosis and hepatomegaly.

**Table 1:** Summary of the reported cases with NPC having pulmonary manifestation/s

Exon/Intron	No. of cases	Mutations Reported			Ethnic origins	References
		Change at Nucleotide level	Change at Protein level	Types of mutation		
Exon 1	*N* = 6	c.58G > T/c.58G > T	p.E20X/p.E20X	Nonsense	French (*n* = 1), Algerian (*n* = 1), Italian (*n* = 2), Czech (*n* = 1)	16, 18
		c.58G > T/c.27delG	p.E20X/p.L9SfsX	Frameshift	French (*n* = 1)	18
Intron 1	*N* = 2	c.82 + 2 T > C /c.82 + 2 T > C	NA	Splice site	Sri-Lanka (*n* = 1) Indian (*n* = 1)	16, Present study
Exon 2	*N* = 4	c.133C > T/c.133C > T	p.Q45X/p.Q45X	Nonsense	NA (*n* = 1)	11
		c.141C > A/c.141C > A	p.C47X/p.C47X	Nonsense	NA (*n* = 1) Indian (*n* = 1)	11, Present study
		c.165C > G/c.165C > G	p.Y55X/p.Y55X	Nonsense	Pakistani (*n* = 1)	11
Exon 3	*N* = 4	c.199 T > C/c.199 T > C	p.S67P/p.S67P	Missense	Turkish (*n* = 1)	18
		c.332delA/c.332delA	p.N111IfsX/p.N111IfsX	Frameshift	NA (*n* = 1)	3, 15
		c.352G > T/c.352G > T	p.E118X/p.E118X	Nonsense	German (*n* = 2)	18, 19
Intron 3	*N* = 1	IVS3 + 6 T > G	EX3del/fs75X	Spliceing	NA	21
Exon 4	*N* = 2	c.434 T > A/c.434 T > A	p.V145E/p.V145E	Missense	Turkish (*n* = 1)	22
		c.436C > T/c.436C > T	p.Q146X/p.Q146X	Nonsense	Tunisian (*n* = 1)	13

*NA -* Not available

Reporting more cases of *NPC2* having pulmonary phenotype will help the clinicians to reach at an early diagnosis and counselling to the family for possible therapeutic options. Though Miglustat therapy is known to be useful for NPC disease with neurological disease but not with pulmonary manifestations [22] successful allogeneic bone marrow transplant [23] was performed in one patient with *NPC2* and may be helpful in further patients once early diagnosis is clinched.

## Conclusion

Mutations in *NPC2* gene were studied for the first time in Indian subcontinent and rarely been reported in the literature. Accurate recognition of young patients with primary unexplained alveolar fibrosis/proteinosis together with hepatomegaly suggesting NPC with molecular characterization of *NPC2* gene is essential. This will provide important new information about functional domains of *NPC2* and advance the understanding of its precise function and its potential interactions with *NPC1.* Moreover, it will enhance our knowledge regarding the underlying pathogenic mechanism responsible for pulmonary manifestation.

## Abbreviation

HRCT-scan: High Resolution Computer Tomography Scan
NPC: Niemann–Pick disease type C
OPD: Out Patient Department
PAS: Periodic Acid Schiff
